# Prevalence of and Factors Associated with Smoking among Japanese Medical Students

**DOI:** 10.2188/jea.JE20090127

**Published:** 2010-07-05

**Authors:** Tetsuo Tamaki, Yoshitaka Kaneita, Takashi Ohida, Eise Yokoyama, Yoneatsu Osaki, Hideyuki Kanda, Shinji Takemura, Kenji Hayashi

**Affiliations:** 1 Department of Public Health, Nihon University School of Medicine, Tokyo, Japan; 2 Division of Environmental and Preventive Medicine, Department of Social Medicine, Tottori University Faculty of Medicine, Tottori, Japan; 3 Department of Hygiene and Preventive Medicine, Fukushima Medical University, Japan; 4 Department of Public Health Policy, National Institute of Public Health, Wako, Japan

**Keywords:** smoking behavior, Japanese medical students, Japan

## Abstract

**Background:**

The aim of the present study was to determine the prevalence of and factors associated with smoking among Japanese medical students, to help promote effective antismoking measures in this population.

**Methods:**

From the 80 university medical schools in Japan, 20 were randomly selected and invited to participate in our survey. The survey focused on medical students and employed an anonymous self-administered questionnaire. Information on each university’s antismoking measures was obtained using a separate questionnaire administered to teaching staff. The survey was conducted from December 2006 through March 2007. Factors associated with smoking were identified by using the chi-square test and multiple logistic regression analysis.

**Results:**

A total of 1619 valid surveys were returned. The overall prevalence of smoking was 13.7% (18.1% among men and 5.1% among women). Factors associated with smoking among medical students were male sex, enrollment at a private medical university, smoking by siblings, alcohol consumption, coffee consumption, insomnia, and less than 6 hours of sleep per night.

**Conclusions:**

Antismoking education must be further promoted to Japanese medical students, with consideration given to the factors associated with smoking behavior found in the present study.

## INTRODUCTION

In 1999, the World Health Organization (WHO) advanced the position that physicians—as role models of healthy living—should not smoke, and that they should not overlook smoking in their patients.^1^ It has been reported that smoking-cessation guidance and intervention by physicians have a significant effect on patients’ smoking behavior. Accordingly, greater importance has been placed on antismoking attitudes among physicians.^2^

It was in this context that, in 2000 and 2004, the Japan Medical Association conducted epidemiological surveys of smoking behavior among its members, and of smoking-cessation interventions that physicians directed toward their patients. The results showed that the prevalence of smoking was 27.1% and 21.5% among male physicians in 2000 and 2004, respectively, and 6.8% and 5.4% among females.^3^ The prevalence of smoking among physicians was lower than that in the general adult population in Japan (43.3% among men and 12.0% among women in 2006); however, it was noted that the prevalence was higher than that among physicians in the United States (ie, approximately 5%).^4^

Health care professionals, including physicians, are believed to be aware of the adverse effects of smoking on health because they are frequently exposed to the relevant data and research in this field. Therefore, it is very important to determine the prevalence of smoking among medical students, the views of this population on smoking among patients, and the nature of antismoking education provided in medical schools. This knowledge would be of benefit in preventing future physicians from smoking. Numerous studies have investigated smoking and its associated factors among medical students, including those that address prevalence, smoking within their families, and attitudes towards antismoking measures. These studies have been conducted in and outside Japan,^5^^–^^9^ although no study in Japan has included a representative sample of medical students. Thus, we investigated smoking habits, and factors associated with smoking, in medical students enrolled at randomly selected medical schools across Japan.

## METHODS

Among the 80 medical schools in Japan, 20 were randomly selected and invited to participate in our survey (November 2006). All of these institutions were willing to cooperate and were sent the study questionnaires with return envelopes. University teaching staff were requested to explain the purpose and outline of the study to the medical students, and to ensure that it was clear that participation was voluntary. The privacy protection policy for personal and enrollment data was clearly stated in the questionnaire. The study was approved by the Ethics Committee of the National Institute of Public Health.

The survey was conducted from December 2006 through March 2007. In all schools, the participants were 4th-year medical students. Participants returned the completed anonymous self-administered questionnaire to teaching staff in a sealed envelope. The teaching staff completed a separate questionnaire regarding the antismoking measures taken at their university.

The survey items for students were as follows: demographic characteristics (sex and age), current smoking status, smoking status in the past, 8 items from the Japanese version of the Fagerstrom Tolerance Questionnaire (FTQ)^10^^–^^13^ (associated with nicotine dependence), views on smoking, smoking in their family, alcohol consumption, coffee consumption, participation in club activities, exercise habits, sleep status, items from the Epworth Sleepiness Scale (ESS)^14^ addressing daytime sleepiness, and mental health, assessed with the General Health Questionnaire (GHQ).^15^ The survey items for university teaching staff were as follows: smoking restriction in the university, tobacco sales on campus, and the status of health education implementation, including that pertaining to smoking (the primary questions addressing smoking and the response options in the student questionnaire are shown in the Appendix).

Participants who answered the question on current smoking status with either “daily” or “sometimes” were defined as smokers; all others were defined as nonsmokers. Participants with an FTQ score of 4 points or higher were defined as nicotine-dependent. Questions regarding sleep status included items addressing difficulty in initiating sleep, difficulty in maintaining sleep, and early-morning awakening: a participant with 1 or more of these symptoms was defined as having insomnia.

We determined smoking prevalence in relation to each item of the survey, and used the chi-square test and multiple logistic regression analysis to ascertain factors associated with smoking. In the multiple logistic regression analysis, current smoking status was considered as a dependent variable, and the following items as independent variables: type of university (national/public or private), demographic characteristics (age and sex), smoking status of family members (parents and siblings), alcohol consumption, coffee consumption, participation or nonparticipation in club activities, physical exercise, presence or absence of sleep disorders, daytime sleepiness score, and GHQ score. The adjusted odds ratios (ORs) for smoking, and their 95% confidence intervals (95% CIs), were calculated. All statistical analyses were performed using SPSS for Windows Version 11.5, with a significance level of *P* < 0.05.

## RESULTS

### Participant characteristics

We invited 1900 medical students (male: 1287; female: 613) to participate in the study, of whom 1683 responded (collection rate: 88.6%). After excluding those who failed to indicate their age (*n* = 15), sex (*n* = 10), or both (*n* = 22), and those returning blank questionnaires (*n* = 17), data for 1619 participants (male: 1074 [66.3%]; female: 545 [33.7%]) were available for analysis (Table 1).

**Table 1.** Characteristics of participants

Age (years)	Male	Female	Total
21–24	880 (81.9%)	484 (88.8%)	1364 (84.2%)
25–29	154 (14.3%)	46 (8.4%)	200 (12.4%)
30–	40 (3.7%)	15 (2.8%)	55 (3.4%)

Total	1074 (100.0%)	545 (100.0%)	1619 (100.0%)

Data were collected from December 2006 through March 2007.

### Prevalence of smoking

The prevalence of smoking in relation to each survey item is shown in Table 2. The prevalence of smoking among men was significantly higher than among women (18.1% vs 5.1%); it was also significantly higher among private university students than among national/public university students (20.7% vs 10.9%).

**Table 2.** Prevalence of smoking among Japanese medical students

	*n*	95% CI		*n*	95% CI
Total	1619 (13.7%)	12.0–15.4	Parent smoker (*n* = 1613)		
			No	1149 (12.6%)	10.7–14.5
Sex (*n* = 1619)			Yes	464 (16.6%)	13.2–20.0
Male	1074 (18.1%)	15.8–20.4			
Female	545 (5.1%)	3.3–6.9	Sibling smoker (=1617)		
			No, or no sibling	1272 (11.2%)	9.5–12.9
Age (years) (*n* = 1619)			Yes	345 (22.9%)	18.5–27.3
21–24	1364 (12.0%)	10.3–13.7			
25–29	200 (24.5%)	18.5–30.5	Drinking alcohol (*n* = 1617)		
30–	55 (16.4%)	6.6–26.2	No	1291 (10.8%)	9.1–12.5
			Yes	326 (24.8%)	20.1–29.5
Type of university (*n* = 1619)					
National/public	1161 (10.9%)	9.1–12.7	Drinking coffee (*n* = 1619)		
Private	458 (20.7%)	17.0–22.4	No	761 (9.6%)	7.5–11.7
			Yes	858 (17.4%)	14.9–19.9
Smoking restriction (*n* = 1619)					
Total smoking ban	517 (13.3%)	10.4–16.2	Participate in school club activities (*n* = 1617)		
Designated smoking area	1102 (13.9%)	11.9–15.9	No	347 (16.7%)	12.8–20.6
			Yes	1270 (12.9%)	11.1–14.7
Cigarette vending machines (*n* = 1538)					
No	1353 (14.5%)	12.6–16.4	Exercise (*n* = 1619)		
Yes	185 (6.5%)	2.7–9.7	No	978 (13.8%)	11.6–16.0
			Yes	641 (13.6%)	10.9–16.3
Sale of cigarettes in campus store (*n* = 1302)					
No	1031 (14.0%)	11.9–16.1	Insomnia (*n* = 1619)		
Yes	271 (13.7%)	9.6–17.8	No	1332 (12.1%)	10.3–13.9
			Yes	287 (21.3%)	16.6–26.0
Passive smoking exposure (days/wk) (*n* = 1540)					
0–3	1208 (7.4%)	5.9–8.9	Sleep duration (hours; *n* = 1616)		
4–7	332 (33.4%)	28.3–38.5	6>	350 (17.1%)	13.2–21.0
			6≦	1266 (12.8%)	11.0–14.6
Did you learn about smoking at school? (*n* = 1609)					
Yes	1298 (13.4%)	11.5–15.3	ESS (Daytime sleepiness) (*n* = 1562)		
No	311 (15.4%)	11.4–19.4	11>	1362 (13.7%)	11.9–15.5
			11≦	200 (12.0%)	7.5–16.5
					
			GHQ (*n* = 1600)		
			4>	949 (13.9%)	11.7–16.1
			4≦	651 (13.4%)	10.8–16.0

Data were collected from December 2006 through March 2007.

For each item, the subjects who did not answer the related questions were excluded.

ESS: Epworth Sleepiness Scale.

GHQ: General Health Questionnaire.

### Factors associated with smoking

The results of logistic regression analysis are shown in Table 3. Multivariate analysis revealed significantly higher adjusted ORs for smoking in relation to male sex, enrollment in a private university, smoking by siblings, alcohol consumption, coffee consumption, insomnia, and less than 6 hours of sleep per night.

**Table 3.** Results of logistic regression analysis of smoking among Japanese medical students

	Crude OR	95% CI	*P*	Adjusted OR	95% CI	*P*
Sex			<0.001			<0.001
Male	1.00	reference		1.00	reference	
Female	0.25	0.16–0.37		0.29	0.18–0.48	

Age (years)			<0.001			0.480
21–24	1.00	reference		1.00	reference	
25–29	2.37	1.66–3.41		1.35	0.81–2.24	
30–	1.43	0.69–2.98		0.86	0.30–2.42	

Type of university			<0.001			0.031
National/public	1.00	reference		1.00	reference	
Private	2.13	1.59–2.85		1.83	1.06–3.16	

Smoking restriction			0.769			0.675
Total smoking ban	1.00	reference		1.00	reference	
Designated smoking area	0.96	0.70–1.30		1.12	0.65–1.94	

Cigarette vending machines			0.004			0.169
Yes	1.00	reference		1.00	reference	
No	0.41	0.22–0.75		0.52	0.21–1.32	

Sale of cigarettes in campus store			0.894			0.581
Yes	1.00	reference		1.00	reference	
No	0.97	0.66–1.44		0.83	0.43–1.61	

Parent smoker			0.036			0.414
No	1.00	reference		1.00	reference	
Yes	1.38	1.02–1.86		1.18	0.80–1.75	

Sibling smoker			<0.001			<0.001
No, or no siblings	1.00	reference		1.00	reference	
Yes	2.35	1.73–3.18		2.08	1.40–3.09	

Drinking alcohol			<0.001			<0.001
No	1.00	reference		1.00	reference	
Yes	2.72	2.00–3.69		2.38	1.62–3.50	

Drinking coffee			<0.001			<0.001
No	1.00	reference		1.00	reference	
Yes	1.98	1.47–2.67		2.36	1.59–3.50	

Participate in school club activities			0.069			0.247
No	1.00	reference		1.00	reference	
Yes	0.74	0.53–1.02		0.77	0.49–1.20	

Exercise			0.895			0.168
No	1.00	reference		1.00	reference	
Yes	0.98	0.73–1.31		0.76	0.52–1.12	

Insomnia			<0.001			<0.001
No	1.00	reference		1.00	reference	
Yes	1.96	1.42–2.72		2.16	1.38–3.40	

Sleep duration (hours)			0.037			0.030
6>	1.41	1.02–1.95		1.59	1.05–2.41	
6≦	1.00	reference		1.00	reference	

ESS (Daytime sleepiness)			0.504			0.382
11>	1.00	reference		1.00	reference	
11≦	0.86	0.54–1.35		0.77	0.43–1.38	

GHQ			0.755			0.725
4>	1.00	reference		1.00	reference	
4≦	0.96	0.71–1.28		0.93	0.63–1.39	

Data were collected from December 2006 through March 2007.

For each item, the subjects who did not answer the related questions were excluded from analyses.

ESS: Epworth Sleepiness Scale.

GHQ: General Health Questionnaire.

### Nicotine dependence

The percentages of respondents classified as nicotine-dependent are shown in Table 4; this factor was associated with male sex, age 25–29 years, and enrollment in a private university.

**Table 4.** Nicotine dependence rate

	*n*	95% CI	*P*
Total	222 (43.2%)	36.7–49.7	

Sex			0.037
Male	194 (45.9%)	38.9–52.9	
Female	28 (25.0%)	9.0–41.0	

Age (years)			0.002
21–24	164 (37.2%)	29.8–44.6	
25–29	49 (65.3%)	52.0–78.6	
30–	9 (33.3%)	2.5–64.1	

Type of university			0.030
National/public	127 (37.0%)	28.5–45.5	
Private	95 (51.6%)	41.6–61.6	

Data were collected from December 2006 through March 2007.

We used 8 items from the Japanese-language version of the Fagerstrom Tolerance Questionnaire; dependence was defined as a score of ≥4 points.

### Age at smoking initiation and attitudes towards smoking cessation among smokers

More than 70% of the smokers had begun smoking before age 20 years, 66.7% had attempted to quit smoking in the past, 53.8% wished to quit, and 56.4% had been advised to quit (Table 5).

**Table 5.** Age at smoking initiation and attitudes towards smoking cessation among smokers

	*n*
Age at smoking initiation (years) (*n* = 202)	
10>	3 (1.5%)
10–19	145 (71.8%)
20≦	54 (26.7%)

Attempted to quit smoking (*n* = 207)	
No	69 (33.3%)
Yes	138 (66.7%)

Motivated to quit smoking (*n* = 195)	
No	90 (46.2%)
Yes	105 (53.8%)

Advised to quit smoking (*n* = 195)	
No	85 (43.6%)
Yes	110 (56.4%)

Data were collected from December 2006 through March 2007.

For each item, the subjects who did not answer the related questions were excluded.

### Antismoking measures taken on campus

At the time of the survey, in 2006, the number of universities that permitted smoking in some areas of the campus exceeded the number of those that prohibited smoking throughout the campus. Seventeen of the 19 schools (the answer was unclear for 1 school) reported having no cigarette-vending machines on campus and no campus stores where tobacco was sold. Health education concerning tobacco was provided by 17 of the 19 schools (the answer was unclear for 1 school; Table 6).

**Table 6.** Antismoking measures taken on campus

	Smoking restriction	
	
	Total smoking ban (*n* = 7; 35%)	Designated smoking area (*n* = 13; 65%)
		
	*n*	*n*
Cigarette vending machines (*n* = 19)		
No	7 (36.8%)	10 (52.6%)
Yes	0 (0.0%)	2 (10.5%)

Sale of cigarettes in campus store (*n* = 16)		
No	6 (37.5%)	7 (43.7%)
Yes	0 (0.0%)	3 (18.8%)

Health education concerning smoking (*n* = 19)		
No	0 (0.0%)	2 (10.5%)
Yes	6 (31.6%)	11 (57.9%)

Data were collected from December 2006 through March 2007.

No university had both cigarette vending machines and a campus store that sold cigarettes.

For each item, the subjects who did not answer the related questions were excluded.

## DISCUSSION

In Japan, there are 80 medical schools. The total number of examinees from these schools who registered for the 102nd National Examination for Medical Practitioners (ie, students who were 1 year senior to the present study participants) was 8535, and the male-to-female ratio was 65.5%:34.5%. The number and male:female ratio of the present survey participants were very similar to those of the students who took the 102nd national examination, despite the fact that we sampled only one-fourth of the total number of medical schools nationwide. Furthermore, the 20 medical schools included in this study were randomly selected. These factors suggest that the present study sample was representative of all 4th-year medical students in Japan.

In 2000, a survey of medical students attending private medical schools in Japan found a smoking prevalence of 27.1% (male: 36.7%, female: 10.4%).^16^ A fact-finding survey of smoking conducted by a national university in 2003 found that 15.4% of male and 3.8% of female medical students smoked.^6^ Another survey conducted between 1999 and 2004 found that the prevalence of smoking among male and female medical students at a public medical school was 16.2% and 4.3%, respectively.^7^ In the present survey, the prevalence of smoking among medical students was 13.7% overall—18.1% for males and 5.1% for females. Any direct comparison of these observations should be avoided, however, since the definitions and analytical methods employed in these surveys were different. Nonetheless, it appears that although the prevalence of smoking among medical students (both men and women) has decreased over the last decade, it has remained unchanged over the past few years.

In all previous surveys of medical students, the prevalence of smoking was lower than that among the general adult population (male: 39.9%, female: 10.0%).^17^ However, as compared with the recently reported prevalence of smoking among medical students in Western countries (eg, 2% in the United States and 4% to 5% in Australia), the prevalence among medical students in Japan is certainly high.^9^

The present study found that more than 60% of the medical students who currently smoked had attempted to quit smoking in the past, and that more than 50% of them wished to quit (Table 5). However, given that they were still smoking, we infer that many of these students failed to quit smoking because of nicotine dependence. In fact, more than 60% of the smokers we surveyed had FTQ scores consistent with nicotine dependence.

Our multivariate analysis revealed that the adjusted odds ratio for smoking was higher in respondents who reported having siblings who smoked. A close association between smoking initiation in adolescents and the smoking behavior of their family members has been demonstrated previously.^18^ Our study shows that, in this regard, medical students are no exception. As for alcohol and coffee consumption, the odds ratio for smoking was higher for students who consumed alcohol or coffee, as compared with those who did not. An association between smoking and alcohol consumption has been demonstrated previously for population samples other than physicians and medical students. Indeed, it is clear that alcohol consumption is a factor strongly associated with smoking behavior.^19^^,^^20^ An association between smoking and coffee consumption is also well documented: a relationship between caffeine intake and the number of cigarettes smoked daily has been reported.^21^ The present study found that the factors associated with smoking among medical students were no different from those found in the general population.

Previous studies on the association between smoking and sleep disorders found that smokers had difficulty in initiating and maintaining sleep.^22^^,^^23^ The stimulant effect of nicotine intensifies with increased nicotine intake and can persist for several hours.^22^ Thus, smoking before going to bed is thought to hinder sleep onset. The stimulant effect of nicotine may contribute to the associations between sleep problems and smoking observed in the present study.

Approximately 90% of the universities we surveyed provided antismoking education for their medical students, and around 80% of students indicated that they had attended such lectures. Nevertheless, it seems that activities aimed at antismoking education, and information on the adverse effects of smoking, have little effect on some students, who nevertheless continue to smoke. Thus, health education programs that are more effective in promoting behavioral change among students are required.

The present study found no association between the prevalence of smoking among medical students and the presence/absence of a campus-wide smoking ban or the sale of tobacco on campus (including the university hospital) from vending machines or in campus stores. However, as indicated by a previous study,^24^ the prevalence of smoking among medical students may have gradually decreased over time after a total ban on smoking was implemented in medical schools.

In our study, the period after the ban on smoking on campus was implemented at each university was not included in our analysis because, in some universities, the ban may not have been completely effective in decreasing the prevalence of smoking because the period of implementation was short. In addition, some students may have smoked on campus, thereby violating the enforced ban, while other students may have smoked in smoking areas in an adjacent hospital or other adjacent facilities.

We believe that medical schools, which train the physicians who will play a central role in providing health care services, must ban smoking on their premises and provide antismoking education to their students for disease prevention.

### Study limitations

The present study had some limitations. First, we only surveyed 4th-year medical students, ie, we did not establish whether there was a difference in the prevalence of smoking across academic years, or whether the prevalence of smoking changed before and after the provision of antismoking education at any stage during the 6 years of medical school attendance. The second limitation was that smoking behavior was assessed on the basis of self-reported data only, without the use of biochemical measurements. The survey participants may have underestimated their own smoking behavior to appear to adhere to the widely accepted idea that medical students should not smoke. Finally, the study’s cross-sectional design does not allow for the determination of causal relationships between smoking behavior and associated factors. Therefore, the factors found to be associated with smoking may not all be risk factors.

**Appendix.** Primary questions addressing smoking, and their response options, in the student questionnaire

1.	Have you ever smoked even 1 cigarette? [No/Yes]
2.	Have you ever smoked daily for 6 months or more? [No/Yes]
3.	Do you currently smoke? [daily/sometimes/never]
4.	(For those who answered “daily” or “sometimes” to Question 3) (1) Have you ever abstained from smoking? [No/Yes] (2) Do you currently wish to quit smoking? [No/Yes] (3) Have you been advised to quit smoking? [No/Yes] (4) Have you smoked during the past year in the buildings of the university? [No/Yes] (5) Have you smoked during the past year on the premises of the university? [No/Yes]
5.	(To those who answered “daily” to Question 3) How many cigarettes do you smoke daily?
6.	How many days have you experienced passive smoking over the past 7 days?
7.	In view of your position as a medical student, do you think that you should not smoke? [No, I do not think it is a problem/Yes, I think I should not smoke/I don’t know]
8.	What is your view on smoking by patients? [They should not smoke/There is no problem with them smoking/Patients may smoke at their discretion/I don’t know]
9.	For the following questions, please answer “No” or “Yes” to express your opinion.
	1) We should make it a policy not to smoke in public. [No/Yes]
	2) A doctor should master the ability to be a non-smoker. [No/Yes]
	3) A doctor should be an example to patients with regard to cessation of smoking. [No/Yes]
	4) A doctor should advise patients not to smoke. [No/Yes]
	5) A doctor should provide patients with information related to non-smoking. [No/Yes]
	6) If a doctor quits smoking, his/her patients will find it easier to quit smoking. [No/Yes]
	7) A doctor is as free to smoke as people in other occupations. [No/Yes]
10.	Did you learn about smoking at school? [No/Yes]
11.	Does your father smoke? [He doesn’t smoke/He used to smoke/He smokes currently/I have no father]
12.	Does your mother smoke? [She doesn’t smoke/She used to smoke/She smokes currently/I have no mother]
13.	Do you have a brother who smokes? [They don’t smoke/They used to smoke/They smoke currently/I have no brothers]
14.	Do you have a sister who smokes? [They don’t smoke/They used to smoke/They smoke currently/I have no sisters]
